# Gas induced formation of inactive Li in rechargeable lithium metal batteries

**DOI:** 10.1038/s41467-022-35779-0

**Published:** 2023-01-12

**Authors:** Yuxuan Xiang, Mingming Tao, Xiaoxuan Chen, Peizhao Shan, Danhui Zhao, Jue Wu, Min Lin, Xiangsi Liu, Huajin He, Weimin Zhao, Yonggang Hu, Junning Chen, Yuexing Wang, Yong Yang

**Affiliations:** 1 State Key Laboratory for Physical Chemistry of Solid Surfaces, Collaborative Innovation Center of Chemistry for Energy Materials and Department of Chemistry, College of Chemistry and Chemical Engineering, Xiamen University, 361005 Xiamen, China; 2 School of Engineering, Westlake University, Hangzhou, 310030 Zhejiang China; 3 College of Chemical Engineering and Safety, Binzhou University, 256603 Binzhou, China; 4 Institute of Electronic Engineering, China Academy of Engineering Physics, 621999 Mianyang, China

**Keywords:** Batteries, Mass spectrometry, Batteries, Batteries

## Abstract

The formation of inactive lithium by side reactions with liquid electrolyte contributes to cell failure of lithium metal batteries. To inhibit the formation and growth of inactive lithium, further understanding of the formation mechanisms and composition of inactive lithium are needed. Here we study the impact of gas producing reactions on the formation of inactive lithium using ethylene carbonate as a case study. Ethylene carbonate is a common electrolyte component used with graphite-based anodes but is incompatible with Li metal anodes. Using mass spectrometry titrations combined with ^13^C and ^2^H isotopic labeling, we reveal that ethylene carbonate decomposition continuously releases ethylene gas, which further reacts with lithium metal to form the electrochemically inactive species LiH and Li_2_C_2_. In addition, phase-field simulations suggest the non-ionically conducting gaseous species could result in an uneven distribution of lithium ions, detrimentally enhancing the formation of dendrites and dead Li. By optimizing the electrolyte composition, we selectively suppress the formation of ethylene gas to limit the formation of LiH and Li_2_C_2_ for both Li metal and graphite-based anodes.

## Abstract (web summary)

The formation of electrochemically inactive, or “dead”, lithium limits the reversibility of lithium metal batteries. Here the authors elucidate the (electro)chemical roles of ethylene gas produced from electrolyte decomposition on the formation of inactive lithium.

## Introduction

The interfacial properties dictate the various functionalities of electrochemical systems. The successful commercialization of Li-ion batteries (LiBs) benefits from successfully addressing the interfacial compatibility problem between graphite anode and electrolytes^1^. However, graphite-based LiBs are approaching their limits and cannot meet the ever-increasing of energy demand for portal electronics and vehicles^2,3^. Lithium metal anode provides the highest theoretical capacity and lowest potential (−3.04 V versus the standard hydrogen electrode), but the interfacial incompatibility between Li metal and liquid electrolyte would lead to violent side-reactions, which convert active Li to dead Li metal and solid electrolyte interphase (SEI) and further results in fast capacity decay of lithium metal batteries (LMBs)^4,5^. Exploring the composition, functions and formation mechanism of these inactive Li species is critical to understand the failure modes of LMBs, but is challenging to study with most existing analytical techniques, especially when studying the SEI with complex compositions^6^. The complexity of SEI originates from the multifarious reactions between Li metal with various chemicals in battery systems, due to the unique strong reduction tendency of Li metal. These reactions happen sequentially and/or concurrently in an operating battery system, leading to a hierarchical and time-varying SEI. Since Peled et al. first proposed the concept of SEI in 1979^7^, various techniques, such as Fourier transform infrared spectrometer (FTIR)^8,9^, Raman spectroscopy^10–12^, Nuclear magnetic resonance (NMR)^13–15^ and cryogenic-transmission electron microscopy (cryo-TEM)^16,17^ etc., have been employed, in attempts to create a comprehensive depiction of the SEI. At present, it is generally accepted that the SEI components could be sorted into two parts: organic components (ROLi, RCOCO_2_Li, etc. where R is alkyl group), and inorganic components: Li_2_O, LiOH, Li_2_CO_3_, LiF etc^18,19^. All these species are believed to be formed by the (electro)chemical reactions between solid-state Li metal and liquid-state electrolytes, as "solid-electrolyte interphase" infers. Nevertheless, note that the chemicals within battery systems are not limited to the liquid-state electrolyte and solid-state electrode. The formation of gaseous species, such as hydrogen, ethylene, carbon dioxide (CO_2_) and etc., are also widely reported in lithium-ion batteries^20–23^. However, the reactions between gaseous species and lithium metal in the operating battery are rarely discussed^24^, although many gases would spontaneously react with lithium metal: such as CO_2_^25^ and oxygen (O_2_)^26^.

Recently, several independent studies reported that Li metal would react with hydrogen gas to produce lithium hydride (LiH) in the Li metal batteries, suggesting that attention is beginning to be paid to the gas-involved reactions in an operating battery system. Aurbach et al. first discussed the possibility of Li metal reacting with hydrogen (H_2_) to form LiH at room temperature, supported by FTIR data^27^. Kourkoutis et al., for the first time, experimentally observed the existence of LiH in practical Li metal batteries using cryo-TEM combined with Electron Energy Loss Spectroscopy^28^. They found the amount of LiH is one order of magnitude larger than the hydrogen gas that can be produced by the trace amount of water in the electrolyte and suggested that the reduction of organic solvent also can contribute to the formation of H_2_. However, Lucht et al. demonstrated that the chemical reduction of organic solvents would not produce H_2_ but hydrocarbons, such as ethylene, propylene and ethane etc^29^. Therefore, the direct correlation between LiH and hydrogen gas that formed during cycling is not fully understood. In addition, a question still remains: are there other gases that could react with lithium metal, leading to undiscovered inactive lithium species?

In this article, we first correlate the evolution of LiH with gas formation using operando mass spectrometry (OMS), and show that ethylene is the major continuously formed gas specie during the whole cycling process. Using mass spectrometry titration (MST) techniques with isotopic labeling, we prove that ethylene and Li metal can react to produce LiH and lithium carbide (Li_2_C_2_). This is an undisclosed formation process of LiH and also reveals the formation routine of Li_2_C_2_. To study the mechanism of the gas-mediated inactive lithium formation, we employ phase-field simulations to demonstrate the presence of bubbles would alter the distribution of electric field and ion concentration at the interface, resulting in non-uniform Li metal deposition and dead Li formation. By rationally designing the electrolyte, we demonstrate that suppressing the formation of ethylene can further restrain the formation of LiH and Li_2_C_2_. The generality of this conclusion is also validated for graphite-based anodes.

## Results

### Correlate the formation LiH with gas evolution

The existence of LiH in LMBs has been reported in several work previously^27,28,30,31^, but the formation mechanism of LiH is rarely discussed. For example, Aurbach firstly demonstrated that the fresh Li metal would react with hydrogen to produce LiH^27^. This reaction was further verified by Cui et al. by using mass spectrometry titration (MST) technique^31^. The hydrogen gas formation in LIBs is general thought to stem from the electrochemical decomposition of trace amount of water^20,32^, but the formation process of H_2_ in Li metal batteries is rarely discussed^33^. More importantly, the evolution of gas species and the formation of LiH are not well-correlated during prolonged cycles.

**Fig. 1:**
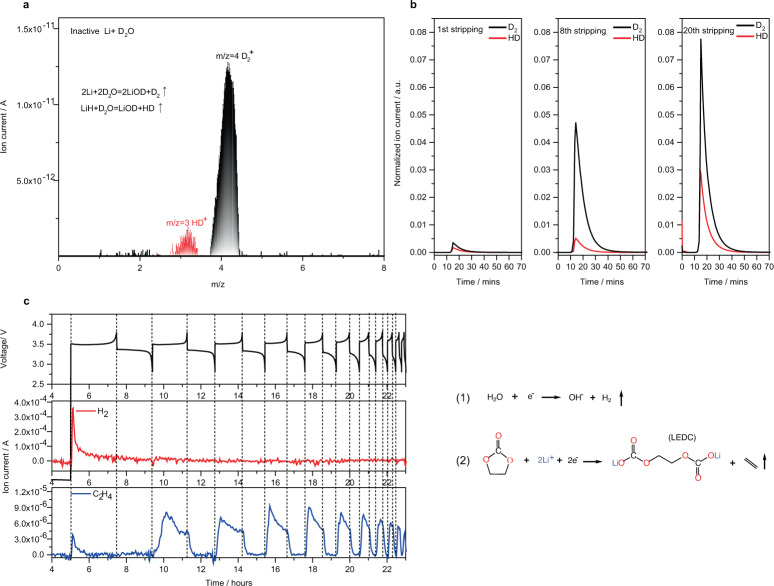
Evolution of LiH and gas species in Cu||LiFePO_4_ batteries using 1 M LiPF_6_/EC:EMC electrolyte. **a** The mass spectrum of gas products produced by reaction between inactive Li with D_2_O. **b** Mass spectrometry titration results of dead Li metal and LiH based on the reaction 2Li + 2D_2_O = 2LiOD + D_2_↑ and LiH + D_2_O = LiOD + HD↑ **c** Voltage profile of Cu||LiFePO_4_ batteries cycled at 0.75 mA cm^−2^ between 2.8 V and 3.8 V, and the corresponding operando mass spectrometry results of H_2_ (m/z = 2) and C_2_H_4_ (m/z = 26).

First, we employ the MST technique as a quantitative method to study the evolution of LiH within inactive lithium that formed in LiFePO_4_||Cu cells using 1 M LiPF_6_/ethylene carbonate (EC):ethyl methyl carbonate (EMC) (Baseline) (The water content of all electrolytes used in this study is <40 ppm). Deuterated water (D_2_O) is the titrant to react with inactive Li to form various gases, among which the HD (half-deuterated hydrogen) gas is solely produced by LiH based on the reaction: LiH + D_2_O = LiOD + HD↑. The HD signal presents a unique mass-to-charge ratio (m/z) value at 3 in the mass spectrum, thus, can be used to track the evolution of LiH (Fig. 1a). Meanwhile, D_2_O reacts with dead Li metal to produce D_2_ (all deuterated hydrogen, m/z = 4), whose evolution represents the dead Li metal formation. Figure 1b shows that the integral area of HD and D_2_ signals both increase continuously with prolonged cycle number, manifesting their irreversible accumulation which causes the continuous loss of active Li. To correlate the formation of LiH with H_2_ gas, we adopt operando mass spectrometer (OMS) to monitor the gas evolution, including H_2_ (m/z = 2), ethylene (C_2_H_4_) (m/z = 28), ethane (C_2_H_6_) (m/z = 30) and CO_2_ (m/z = 44), in anode-free batteries using baseline electrolyte (Fig. 1c and Fig. S1). Of note, H_2_ formation is mainly observed during the first charge process, but is not detected during prolonged cycles (Fig. 1c). This phenomenon is consistent with the reported observation in silicon^22^ anode and Cu||NCM111 batteries^33^, where the H_2_ formation is believed to come from the decomposition of water impurities, thus only dominates in the initial cycles. Based on the assumption that LiH is produced by the reaction between Li metal and hydrogen, this evolution pattern of H_2_ cannot rationalize the continued formation of LiH during the whole cycling process as observed in MST results, suggesting an unclosed formation mechanism of LiH formation during the prolonged cycles.

In contrast to H_2_, we observed a continued evolution of C_2_H_4_ during the whole cycling process (Fig. 1c). For every single cycle, the C_2_H_4_ signal starts to rise during charging (Li deposition) and suddenly decays when the current reverse, suggesting the formation of C_2_H_4_ is accompanied by Li deposition. This gas-evolution pattern is similar to the observation reported for silicon anode using 1M LiPF_6_/EC:EMC electrolyte^22^. A recent study demonstrates the formation of C_2_H_4_ is even more significant than H_2_ in Cu||NCM111 batteries^33^. It is well-documented that C_2_H_4_ is the decomposition product of EC through a two-electrons reduction reaction: EC + 2e^-^ → (CH_2_OCO_2_Li)_2_ + C_2_H_4_^18,29,34^. In addition to C_2_H_4_ observed by OMS, we also saw a continued increase of (CH_2_OCO_2_Li)_2_ through the titration of CO_2_, which is produced by the reactions between D_2_O with (CH_2_OCO_2_Li)_2_ (Fig. S2). The OMS and MST results imply the concurrent process of LiH formation and electrolyte decomposition. Considering the organic electrolyte is the main source of protons, this concurrent process implies a potential correlation between the formation of LiH and the evolution of C_2_H_4_.

### Reaction between Li metal with ethylene

The reaction of Li metal with ethylene was first reported by Guntz et al. in 1896^35^. C_2_H_4_ would react with Li metal under a heating atmosphere to produce grayish-white products, which are analyzed as a mixture of LiH and Li_2_C_2_ based on this equation^36^:

1 $${C}_{2}{H}_{4}+{6{Li}}^{0}\to 4{LiH}+{{Li}}_{2}{C}_{2}$$

However, it is unclear whether this reaction can proceed at room temperature in battery chemistry. We first calculate the Gibbs free energy of this reaction (at 298.15 K), which is to be −398.049 kJ/mol (Supplementary Note 1), indicating this reaction is thermodynamically feasible at room temperature. For reference, the Gibbs free energy of Li metal reacting with H_2_ to produce LiH is −136.894 kJ/mol. More direct evidence for Eq. 1 is using pure Li metal to react with C_2_H_4_ gas at room temperature. Pure Li metal foils were stored in the C_2_H_4_ (99.999%) and Argon (99.999%) atmosphere for 10 days at room temperature. The surface species was then scraped off and analyzed by MST technique. The existence of Li_2_C_2_ can be validated by the signal of acetylene (C_2_H_2_) (m/z = 26) based on the reaction: Li_2_C_2_ + 2H_2_O → 2LiOH + C_2_H_2_. H_2_O rather than D_2_O is used as the titrants so as to avoid the interference of fragments of CO_2_ (CO^+^, m/z = 28) on the analysis of m/z = 28 (C_2_D_2_). Figure S3 shows that the reaction gases of both samples are dominated by H_2_, but importantly, C_2_H_2_ is solely observed for the C_2_H_4_-stored Li metal. This result provides convincible evidence for the feasibility of Eq. 1.

**Fig. 2:**
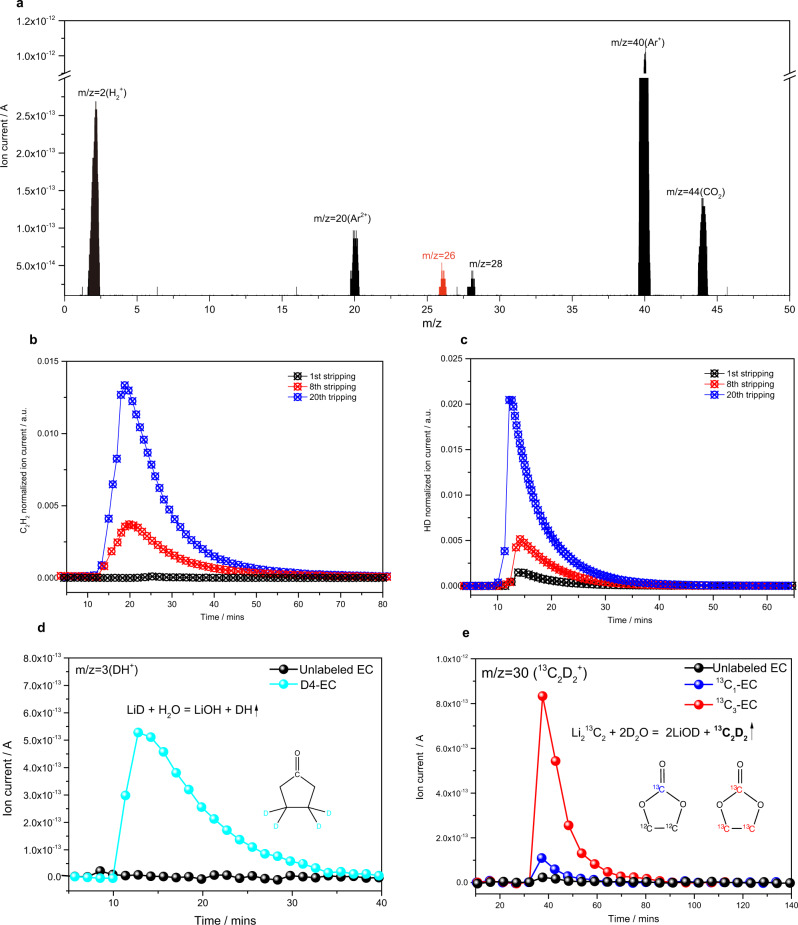
Spontaneous reaction between Li metal with ethylene. **a** Mass spectrometry titration results of inactive Li using H_2_O. The signal C_2_H_2_ (m/z = 26) represents the formation of Li_2_C_2_. The evolution of (**b**) C_2_H_2 _(m/z = 26) signal and (**c**) HD (m/z = 3) signal, representing the accumulation process of Li_2_C_2_ and LiH, respectively. **d** Mass spectrometry titration results of inactive Li formed in unlabeled EC and in all deuterated EC (D_4_-EC). **e** Mass spectrometry titration results of inactive Li formed in unlabeled EC, carbonyl carbon labelled EC (^13^C_1_-EC), and all carbon labelled EC (^13^C_3_-EC).

For an operating battery, LiH is already observed by MST results. The existence of Li_2_C_2_ in inactive Li is still ambiguous, though we have demonstrated the feasibility of Eq. 1. Schmitz et al. first provided spectroscopic evidence of Li_2_C_2_ formed on the surface of Li metal, by observing the Raman signal of acetylene anion^10^. Nevertheless, question remains: How does Li_2_C_2_ evolve during cycling? If Eq. 1 proceeds, does the evolution of Li_2_C_2_ have the same trend as LiH in the operating batteries? Here, we employed MST technique to explore the presence and evolution of Li_2_C_2_ in the inactive Li formed in Cu||LiFePO_4_ batteries. Figure 2a shows a typical titration result of inactive Li formed in baseline electrolyte after 20 cycles using H_2_O titrants. H_2_ (m/z = 2) and CO_2_ (m/z = 44) dominate the reaction gas, corresponding to the formation of dead Li metal (or LiH) and lithium ethylene dicarbonate (LEDC) species. Importantly, the presence of signal C_2_H_2_ (m/z = 26) validates the existence of Li_2_C_2_ after stripping (Fig. 2a), demonstrating its irreversible nature, which has not been discussed in the Li metal batteries. We performed the MST measurements for the inactive Li formed after the 1st, 8th, and 20th cycles and found a similar evolution pattern of Li_2_C_2_ (Fig. 2b) to that of LiH (Fig. 2c). The mutual formation and increase of these two inactive Li species also imply the feasibility of Eq. 1.

To further prove that the formation of LiH and Li_2_C_2_ is directly related to ethylene, a product of EC decomposition, we used three isotope-labeled EC: all deuterated EC (D_4_-EC), carbonyl carbon labelled EC (^13^C_1_-EC) and all carbon labeled EC (^13^C_3_-EC), to replace the natural abundance EC (unlabeled EC) to form LiPF_6_/EC: EMC electrolyte. The inactive Li formed in D_4_-electrolyte and unlabeled electrolyte are titrated by H_2_O. DH^+^(m/z = 3) signal is solely observed in D_4_-electrolyte, confirming the formation of LiD. This result validated that the LiH formation is directly related to the proton in EC solvents. In addition, when using D_2_O to titrate the inactive Li formed in ^13^C_1_ electrolyte and ^13^C_3_ electrolyte, the ^13^C_2_D_2_^+^(m/z = 30) signal is mainly observed for the ^13^C_3_ electrolyte while the signal is small for the ^13^C_1_ electrolyte and unlabeled EC. This result indicates that the Li_2_C_2_ formation is dominantly related to the non-carbonyl carbon, which is also the source of C_2_H_4_ (reaction 2 in Fig. 1).

In addition, we checked all literature that reported the existence of Li_2_C_2_ and found that EC solvents were all used in these reports regardless of the electrolyte formulation changes (Table S1). In addition, cryo-electron microscopy (cryo-EM) results showed that ethylene bubbles and LiH both existed at the lithium metal interface^28^. Such spatial proximity also provides the possibility for the reaction of lithium metal and ethylene. Of note, we cannot completely rule out the possibility that other solvents will produce ethylene. Therefore, other solvents or additives capable of producing ethylene should be used with caution in the future similar studies, as this may lead to the additional formation of LiH and Li_2_C_2_. And other formation mechanisms for LiH and Li_2_C_2_ could be pursued in future study. Just as lithium fluoride (LiF) has diverse formation paths in battery chemistry, which largely depends on the used solvents and additives.

### Impacts of gas on the Li metal deposition

The previous studies using cryo-EM demonstrate that LiH can be found on the lithium dendrite and it is spatially closer to Li dendrite than it is to the organic SEI species^28^. In-depth X-ray photoelectron spectroscopy suggested that the Li_2_C_2_ locates at the inner shell of SEI layer^37,38^. Therefore, the physical properties, including ionic and electronic conductivities, of LiH and Li_2_C_2_ would have a great impact on the interfacial reactions of Li metal anode. We compared the energy barriers of lithium diffusion in common inorganic SEI species (Table S2). The lithium diffusion in Li_2_C_2_ is much slower than that of Li_2_O and Li_2_CO_3_, but comparable with LiF, whose ionic conductivity is extremely small. We then compared the bandgaps of these inorganic SEIs. The bandgap of LiH and Li_2_C_2_ is lower than that of Li_2_O, Li_2_CO_3_ and LiF, suggesting it’s potentially higher electronic conductivities, which is detrimental to the battery performance. (Fig. S4 and Table S2). Therefore, considering the ionic and electronic conductivities, LiH and Li_2_C_2_ are believed to be not ideal SEI species formed on the surface of lithium metal, which may result in the large interfacial impedance and non-uniform Li ion flux.

**Fig. 3:**
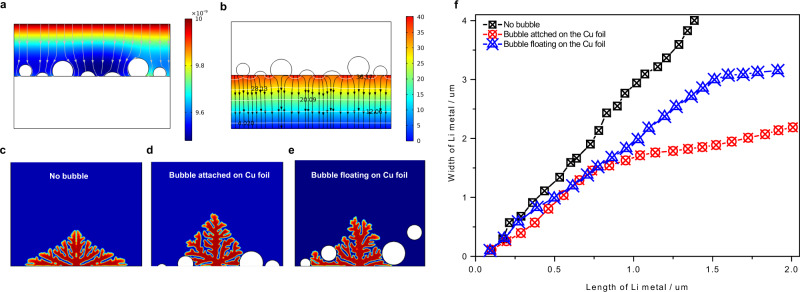
Gas effects on the Li metal deposition. The simulation of the distribution of (**a**) Li^+^ concentration and (**b**) electric field with bubbles attached to the surface of copper surface. The bubble-effects on the morphological evolution of deposited Li metal probed by phased field simulation: (**c**) without bubble, (**d**) with bubble attached on the copper surface and (**e**) floating over the copper foil. **f** The bubble effects on the length and the width of dendrites.

In addition to chemical effects, the mechanical effects of gas on the electrochemical process are widely investigated in the gas-evolving electrode, such as water splitting and CO_2_ electro-reduction. In these systems, bubbles would reduce electrochemical active area and block the ionic transport pathways, but the bubble effect on the Li deposition is rarely discussed in lithium batteries. We first use COMSOL simulation to explore the effects of bubbles on the distribution of Li^+^ concentration and electric field. The details of the simulation method are described in the experimental section. We first model the non-Li^+^ and non-electronic conducting bubbles attached to the current collectors. The simulation results demonstrate that bubbles would lead to uneven distribution of Li^+^ concentration (Fig. 3a) and electric field (Fig. 3b and Fig. S5), which is considered to be the cause of lithium dendrites growth^39^. The phase-field model is then further employed to describe the morphology of deposited Li metal^40–42^. Without bubble effects, the homogeneous distribution of Li^+^ and electric field would lead to the deposited Li metal growing horizontally and vertically at the same time, presenting a mossy-type morphology (Fig. 3c), as validated by optical microscopy^43^. With bubbles attached to the Cu foil, the horizontal growth of deposited metal is greatly suppressed (Fig. 3d). In this case, the deposited lithium metal tends to form dendrites. The same scenario is observed in the case that bubbles floating on the Cu foil (Fig. 3e). This result is in line with the optical observation in sodium metal^44^ (Fig. S6), where the dendrites formation is ascribed to the diminishing electrochemical area caused by bubbles. Figure 3f summarizes the length and width of deposited Li metal, which suggests that the existence and position of non-Li^+^ conducting bubbles would facilitate the formation of dendritic Li metal. This result is consistent with a recent simulation study that discussed the bubble-effects on the graphite, which shows that the bubble would lead to the uneven accumulation of Li metal on the surface of graphite anode^45^. Interestingly, we observed the deposited Li metal would grow on the surface of bubbles (Fig. S7). In this case, the detachment of bubbles would overhang some lithium metal fragments from the bulk and form the dead Li metal. These results hint that the dead Li metal may not only form in the stripping process, but the gas detachment would also contribute to the formation of dead Li metal, which has also been observed by optical microscopy in the sodium metal anode^44^.

### Suppressing the formation of LiH and Li_2_C_2_

**Fig. 4:**
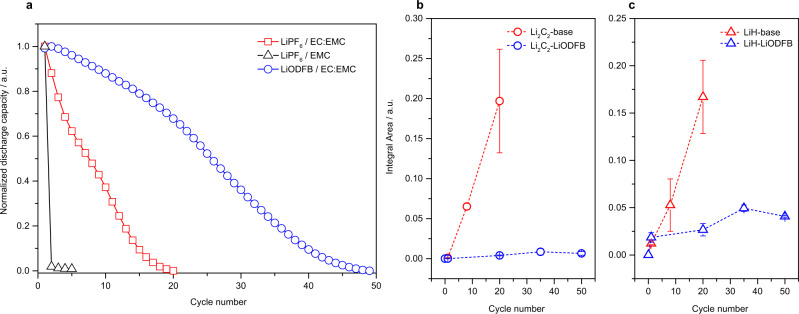
Suppressing LiH and Li_2_C_2_ by electrolyte optimization. **a** The cycle performance of Cu||LiFePO_4_ cells, which cycled between 2.8 V and 3.8 V with a fixed current density of 0.75 mA cm^−2^ using 1 M LiPF_6_/EMC, 1 M LiPF_6_/EC:EMC (baseline) and 1 M LiODFB/EC:EMC (LiODFB) electrolytes. The corresponding titration results of (**b**) Li_2_C_2_ and (**c**) LiH of inactive Li formed in the baseline and LiODFB electrolytes. The error bars come from three independent experiments.

The continuous formation of LiH and Li_2_C_2_ not only results in the active Li loss, but also slow down the interfacial transportation of Li ions. Our results suggest the formation of LiH and Li_2_C_2_ is highly correlated to the evolution of ethylene, which is believed to be mainly from the decomposition of EC. Thus, stunting the formation of ethylene by carefully selecting the electrolyte recipe is the key to preventing the further formation of Li_2_C_2_ and LiH. Firstly, we remove the EC solvents in the baseline electrolyte to form 1 M LiPF_6_/EMC (EMC electrolyte). Though Li_2_C_2_ is not detected in inactive Li for the EMC electrolyte (Fig. S8), AFBs with this EC-free electrolyte lost almost 99% capacity after the first cycle (Fig. 4a, Fig. S9). The reduced formation of Li_2_C_2_ may also come from the limited cycle life. The amount of LiH formed in EMC electrolyte is comparable to that is formed in the baseline electrolyte after 1st cycle (Fig. S10), which could be due to the H_2_ involving in the formation of LiH. This suggests that if we can reduce the water content in the electrolyte or suppress the decomposition of trace water, we can reduce the formation of LiH at the beginning of the cycle. The limited cycle number of EMC electrolyte prevents us from drawing a reliable conclusion. Therefore, we keep the solvents (EC: EMC) unchanged, replacing the LiPF_6_ in the baseline electrolyte with lithium difluoro(oxalato)borate (LiODFB) to form 1 M LiODFB/EC:EMC. LiODFB is prior to decomposed on the Li metal to form so-called anion-derived SEI and prevent the further decomposition of solvents^46^. Figure 4a shows the superior cycling performance of AFBs with LiODFB based electrolyte, demonstrating the protecting effects of LiODFB. Operando mass spectrometry results show the formation of H_2_ dominates in the initial three cycles and then it decays with prolonged cycles, presenting similar behavior in baseline electrolyte (Fig. S11). We integrate the H_2_ area for LiODFB and baseline electrolyte respectively under the same cycling time (20 h), which shows a comparable value (2.25*10^−4^ a.u. for LiODFB and 1.73*10^−4^ a.u.for baseline), indicating a similar amount of H_2_ produced by two different electrolytes. While no evidence of the formation of C_2_H_4_ is detected when using LiODFB electrolyte during the whole cycling process, demonstrating the well-controlled decomposition of EC (Fig. S11). This result can be further validated by the reduced amount of CO_2_ observed through titration, which is the indicator of the decomposition of EC and EMC (Fig. S12). On the basis of the results above and Eq. 1, we can conclude that the EC decomposition is greatly suppressed by using LiODFB.

To validate the above hypothesis, we perform the MST tests to measure the evolution of LiH and Li_2_C_2_ in LiODFB-based electrolytes. Figure 4 shows that the signal of Li_2_C_2_ and LiH both being inhibited when using LiODFB electrolyte, which is consistent with our hypothesis. LiH is still present in LiODFB electrolyte, but rather than the continued increase in baseline electrolyte, LiH formation mainly comes from the first cycle, and increases slowly in the prolonged cycles. This result is in line with the gas evolution in the LiODFB electrolyte: H_2_ dominates in the initial cycles and C_2_H_4_ is suppressed during the whole cycle process.

Furthermore, we completely replace the carbonate solvents by ether-based solvent: 1 M lithium bis-trifluo-romethanesulfonylimide (LiTFSI)/1,3-dioxolane (DOL):1,2-dimethoxyethane (DME) + 2 wt% lithium nitrate (LiNO_3_). The ether-based electrolyte is more stable with Li metal thus less decomposition of electrolyte is expected, which can be evidenced by the stable cycle performance and high Coulombic efficiency (Fig. S13). The MST results indicate the mutual inhibition of LiH and Li_2_C_2_ (Figs. S8d and S10d).

Compared to LMBs, EC solvent is indispensable for commercial LiBs and also would be reduced on the graphite anode to produce C_2_H_4_. Meanwhile, Li plating is also widely reported in graphite anode. Therefore, it is reasonable to surmise that the formation of LiH and Li_2_C_2_ also happens in LiBs. McCloskey et al. observed the formation of Li_2_C_2_ on the graphite anode when using EC-based electrolyte, and hypothesized that the formation of Li_2_C_2_ occurs through the chemical reactions other than electrochemical reactions. By artificially decreasing the N/P to 0.73, we mimic the Li plating in the graphite||LiFePO_4_ cells (Fig. S14). After 20 cycles, the graphite anodes were retrieved and titrated by D_2_O and H_2_O. A similar scenario was observed in graphite anodes: in the LiODFB electrolyte, LiH and Li_2_C_2_ are both inhibited as compared to the baseline electrolyte (Fig. S15).

### Gas induced formation of inactive Li

**Fig. 5:**
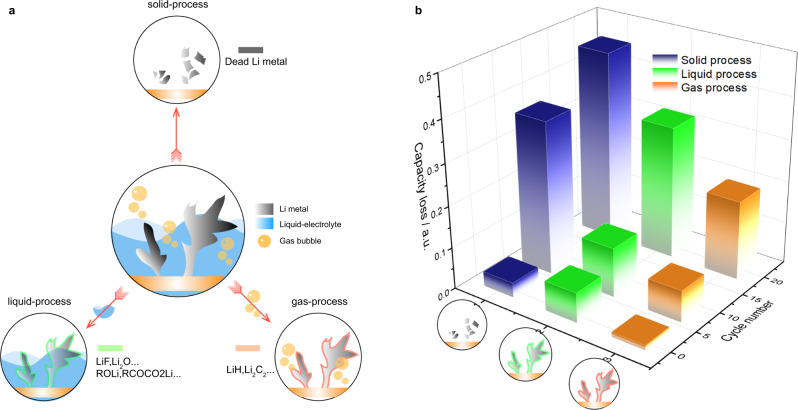
Proposed three formation-process of inactive Li and their corresponding amount that determined by mass spectrometry titration method. **a** The schematic of inactive Li formation through (i) Solid-process: forming dead Li metal; (ii) Liquid-process: forming inactive Li through the reactions between liquid electrolyte with Li metal; (iii) Gas-process: forming inactive Li through the reactions between gas species with Li metal. **b** The inactive Lithium distribution in the baseline electrolyte. We assume the Li_2_C_2_ and LiH are all formed by the chemical reaction between lithium metal and gas species. The rest of inactive lithium (excluding dead Li metal, LiH and Li_2_C_2_) is formed by the reaction between Li metal and liquid electrolyte.

The formation of inactive Li is the ultimate crux of battery failure, which drives research communities to explore the compositions and formation process of inactive lithium, so as to inhibit their formation from the source. The classical SEI model focuses on the solid-liquid two-phase interface. Therefore, the formation of inactive Li generally stems from two processes: (1) solid-process, i.e., the formation process of dead Li metal only involves solids (lithium metal); (2) liquid-process, which describes the SEI formation by the reactions between Li metal with liquid electrolyte (Fig. 5a).

2 $$\begin{array}{cc}{Solid}-{process}\!\!: & {{Li}}^{0}\left(s\right)\to {{Li}}_{{dead}}^{0}\left(s\right).\end{array}$$

3 $$\begin{array}{cc}{Liquid}-{process}\!\!: & {{Li}}^{0}\left(s\right)+{{{{{\rm{electrolyte}}}}}}\left(l\right)\to {{{{{\rm{Inactive}}}}}}\,{{{{{\rm{Li}}}}}}\left(s\right).\end{array}$$

4 $$\begin{array}{cc}{Gas}-{process}\!\!: & {{Li}}^{0}\left(s\right)+{{{{{\rm{gas}}}}}}\left(g\right)\to {{{{{\rm{Inactive}}}}}}\,{{{{{\rm{Li}}}}}}\left(s\right).\end{array}$$

However, the third process: the gas-participate formation of inactive Li is rarely discussed. The gas effects on the formation of inactive Li is first studied by the simulations and calculation method. The molecular dynamics (MD) simulations demonstrated the gas species can be found in the inner shell of SEI layer^47^. Recently, the reactive MD shows that the gas produced by EC decomposition can be found at the bottom part of lithium dendrites. During the lithium stripping process, crystalline lithium atoms can be easily stripped due to these gas species, leading to the formation of dead Li metal^48^. However, this gas-induced formation of dead Li metal is difficult to be validated by the current characterization tools, which requires high spatial resolution (~nm) and sufficient time resolution to observe the very local interaction between gas and Li metal. Recently, cryo-TEM re-examined and depicted the Li metal/electrolyte interface at nano-scale, which evidenced the co-existence of three phases: gas bubbles (ethylene), liquid electrolytes and solid Li metal^28^. Unfortunately, the current cryo-EM technique is a post-mortem analysis, the dynamic interaction between Li metal and gas species is not accessible at present.

The previous results, as least, demonstrated the strong interaction between gas species and lithium metal. In this contribution we disclose and highlight the chemical reactions between lithium metal and gas species (denote as gas-process in Fig. 5a). Taking the ethylene gas as an example, 1 mol ethylene can react with 6 mol Li metal to form inactive Li, while the decomposition of 1 mol EC only produces 0.5 mol inactive Li (LEDC). This stresses the significant effect of gas on the formation of inactive Li. Based on the quantitative capability of mass spectrometry, we differentiate and quantify the inactive Li formed through these three processes. Dead Li metal, LiH and Li_2_C_2_ are quantified by the titration signal of D_2_, HD and C_2_H_2_ respectively. Here, we assume that the Li_2_C_2_ and LiH are totally formed by the chemical reaction between lithium metal and gas species, and the rest inactive Li is formed by the liquid process. The amount of inactive Li formed by the liquid process can be calculated by the equation: total irreversible Li – (dead Li metal) – LiH - Li_2_C_2_. Figure 5b shows the quantitative results of inactive Li formed in Cu||LiFePO_4_ cells using baseline electrolyte. We surprisingly found the contribution of LiH and Li_2_C_2_ to the inactive Li formation is 19.0% of capacity loss after 20 cycles. In contrast, this value is only 3.2% in the LiODFB based electrolyte (Fig. S16); the better cycling performance of the cells in LiODFB based electrolytes can be attributed to the less LiH and Li_2_C_2_ being formed during the whole cycling processes, highlighting the significance of suppressing gas evolution to achieve a longer cycle life in LMBs.

## Discussion

The formation of SEI by decomposition of EC solvent on graphite anode leads to successful commercialization of lithium-ion batteries. However, the incompatibility between EC solvent and lithium metal anode limits the further improvement of battery energy density. In this article, we first disclose that ethylene, a byproduct of EC decomposition, has a similar accumulation pattern with LiH. Using MST and isotope tracing method, we have systematically studied the reactions between Li metal and ethylene, which would contribute to the formation of inactive Li (Li_2_C_2_ and LiH). The phase-filed simulation results illustrate the presence of gas bubbles would exert the morphology of deposited Li metal and result in the more dendrites formation. By rationally choosing the electrolyte formula, we are able to suppress the formation of ethylene, and further restrain the formation of LiH and Li_2_C_2_ in Li metal and graphite anode. Our results highlight a neglected formation route of inactive Li—denote as gas-induced inactive Li, which is rarely discussed and lacks comprehensive investigation in battery research. We believe this work would inspire more new adventures in exploring gas effects on the cycle performance of lithium and also other alkaline metal-based batteries such as rechargeable sodium batteries.

## Methods

### Electrochemical

LiFePO_4_ electrodes were prepared by casting a slurry consist of 90% wt LiFePO_4_, 5 wt% polyvinylidene fluoride binder and 5% wt acetylene black on to Al foil, and dried at 80 °C under vacuum. The dried cathodes were then punched into 14 mm disks. The average material loading was about 11.8 mg cm^−2^. The Cu||LiFePO_4_ coin cells of CR2025 size were assembled in an argon filled glove-box (O_2_ < 0.5 ppm, H_2_O < 0.5 ppm) and cycled with a fixed current density of at room temperature. One piece of celgard (2325) as the separator and 50 µL electrolyte was added for each cell. 1 M LiPF_6_ (purchased from Canrd New Energy Technology Co.,Ltd., 99.9%) was dissolved in the mixture of EC and EMC (3:7 by weight) (purchased from Canrd New Energy Technology Co.,Ltd., 99.99%) as the baseline electrolyte_._ Replacing the LiPF_6_ in baseline with LiODFB (purchased from DoDo Chem, 99.8%) to form 1 M LiODFB/EC:EMC electrolyte. 1 M LiTFSI/DOL:DME (1:1 by volume) + 2 wt.% LiNO_3_ electrolyte was purchased from DoDo Chem.

### Operando mass spectrometry

The OMS was carried out using a quadrupole mass spectrometer (Hiden Analytic Ltd.) and a custom Swagelok-type operando cell with gas inlet and outlet was employed. The operando cells were assembled in glove box (O_2_ < 0.5 ppm, H_2_O < 0.5 ppm), and 14 mm LiFePO_4_ cathode (11.8 mg cm^−2^), 16 mm copper foils and one piece of celgard were used. The operando cell was also cycled with 0.75 mA /cm^−2^ current density between 3.8 V and 2.8 V. High purity argon gas (99.999%) was used as the carrier gas, whose flow rate was controlled by a digital mass flow meter (Bronkhorst). After connecting the cell to the OMS system, it is flushed with Ar for 5 h under operando circuit voltage to allow for a stable m/z signal background. The evolved gases H_2_ (m/z = 2), CO_2_ (m/z = 44), C_2_H_4_ (m/z = 28), and C_2_H_6_ (m/z = 30) were monitored continuously during cycling.

### Mass spectrometry titration

Mass spectrometry titration (MST) was performed on a quadrupole mass spectrometer (Hiden Analytic Ltd.). The cycled Cu||LiFePO_4_ cells were disassembled in a glove box. The copper foil and celgard separator were retrieved and put into a well-sealed 5 ml headspace vessel. After the vessel is connected to the mass spectrometry, high purity Ar flushed the headspace of vessel to remove any impurities from the glove box until the m/z signal is stable. Then D_2_O (99.9%) or deionized H_2_O was injected into the vessel to react with inactive Li, producing various gas products that analyzed by mass spectrometry. For a quantitative analysis, the set of following standards with known mass were titrated and giving the standard curves. The standard samples for D_2_, HD and C_2_H_2_ signals are Li metal (99.9%), LiH (99%) and CaC_2_ (98%) respectively.

### Simulation

All the numerical simulation was performed using the commercial finite-element method software COMSOL Multiphysics. A computational ion transport model was built to study the electric potential distribution at the gas-evolving electrode and the Li^+^ concentration distribution in the adjacent electrolyte. Segmented bubble structures with varied surface contact angles reconstructed from experimental observation were fed to COMSOL. Li^+^ transport was considered driven by both electric field and diffusion flow in the system. The following coupled partial differential equations were solved by adding two physical sub-model nodes of electrostatic and transport of diluted species into the simulation^49^.

5 $${{{{{\bf{E}}}}}}=-\nabla {\varphi }_{{{{{{\rm{ele}}}}}}.}$$

6 $${{{{{\bf{J}}}}}}=-{D}^{l}\nabla {c}_{+}+\nu {c}_{+}{{{{{\bf{E}}}}}}{{{{{\boldsymbol{.}}}}}}$$

7 $$\frac{\partial {c}_{+}}{\partial t}+\nabla {{{{{\bf{J}}}}}}={R}_{i}.$$

Where **E** is the electric field, ${\varphi }_{{{{{{\rm{ele}}}}}}}$ is the electrolyte potential, **J** is the Li^+^ flux vector in the electrolyte constituted by diffusion and migration, ${D}^{l}$ is the Li^+^ diffusivity of the electrolyte, ${c}_{ \!+}$ is the Li^+^ concentration in the electrolyte, ${R}_{i}$ is reaction source term, and ${{{{{\rm{\nu }}}}}}$ is the ionic mobility of Li^+^ in the electrolyte, which was computed from the Nernst-Einstein equation $\nu=\frac{{D}^{l}}{{RT}}$ in this work^50^, where *R* is molar gas constant and *T* is absolute reaction temperature. Domain areas of two 60 μm × 20 μm rectangles were created, where the upper one represented the electrolyte phase with a composition of LiPF_6_/EC: EMC and the lower one represented copper electrode with gas bubbles evolving. The bottom boundaries of two simulation areas were defined by Dirichlet boundary conditions with ${\varphi }_{0}=0$ V and ${c}_{0}=0$ M.

On this basis, a nonlinear phase-field model was built to study the electrode–electrolyte interface motion and the resulting Li-dendrite morphology evolution during the electro-chemical deposition on the electrodes with gas bubbles located at different positions. The total Gibbs free energy density during electroplating on the gas-generated electrode surface can be defined as:

8 $$G=\int\limits_{V}\left[f\left(\xi,{\widetilde{c}}_{i}\right)+\frac{1}{2}\nabla {\widetilde{c}}_{i}\left(r,t\right)\cdot \kappa \nabla {\widetilde{c}}_{i}+{\rho }_{e}\varphi \right]{dV}.$$

Where $f\left(\xi,{\widetilde{c}}_{i}\right)$ denotes the Helmholtz free energy density, in which *ξ* is a non-conserved phase-field order variable, whose value varying continuously from 1 to 0 in the diffuse-interfacial region, corresponding to the physical property converting from the solid to the liquid. The order variable *ξ* was introduced to indicate the evolution of lithium phase morphology. ${\widetilde{c}}_{i}$ is the set of dimensionless concentrations of lithium atom, ${{{{{{\rm{Li}}}}}}}^{+}$ and ${{{{{\rm{P}}}}}}{{{{{{\rm{F}}}}}}}_{6}^{-}$, i.e.,$\left\{\widetilde{c}=\frac{c}{{c}_{s}},{\widetilde{c}}_{+}=\frac{{c}_{+}}{{c}_{0}},{\widetilde{c}}_{-}=\frac{{c}_{-}}{{c}_{0}}\right\}$. The second term on the right-hand side of the equation represents gradient energy density, where *κ* is the gradient energy coefficient, related to the surface energy anisotropy.

9 $$\kappa={\kappa }_{0}\left(1+\varOmega {{\cos }}\left(\omega \theta \right)\right).$$

Where ${\kappa }_{0}$ is the interface energy, $\varOmega$ and $\omega$ are the strength and mode of the anisotropy, respectively, and *θ* is the angle between the normal vector of the interface and the reference direction. ${\rho }_{e}\varphi$ denotes the electrostatic energy density, where $\varphi$ is the electrostatic potential, and ${\rho }_{e}$ is the charge density. *V* is the arbitrary dendrite volume.

At the Li/electrolyte interface, the charge transfer rate of Li deposition was given by the total changes in current rate that driven by the potential differences across the interface, using the Butler–Volmer equation.

10 $${{{{{\bf{I}}}}}}={{{{{{\bf{I}}}}}}}_{a}+{{{{{{\bf{I}}}}}}}_{{{{{{\bf{c}}}}}}}={i}_{0}\left[{{\exp }}\left(\frac{{\alpha }_{a}F\eta }{{RT}}\right)-\exp \left(\frac{-{\alpha }_{c}F\eta }{{RT}}\right)\right].$$

Where **I**_a_ (**I**_c_) is the anodic (cathodic) current, *η* is the overpotential, *i*_0_ is the exchange current, and ${\alpha }_{a}$ (${\alpha }_{c}$) is the anodic (cathodic) charge-transfer coefficient. The $\eta$ is proportional to the electrochemical potential change, which is defined to be the partial molar Gibbs free energy in a multispecies system.

11 $$\eta={\varphi }_{{{{{{\rm{Li}}}}}}}-{\varphi }_{{{{{{\rm{e}}}}}}}-{E}_{{{{{{\rm{eq}}}}}}}=\frac{\triangle \mu }{{nF}}=\frac{1}{{nF}}\mathop{\sum}\limits_{i}\frac{\delta G}{\delta {c}_{i}}.$$

Where ${\varphi }_{{{{{{\rm{Li}}}}}}}$ and ${\varphi }_{{{{{{\rm{e}}}}}}}$ denote the electric potential of Li dendrite and electrolyte respectively, and ${E}_{{{{{{\rm{eq}}}}}}}$ is the equilibrium potential for the electrochemical reaction (0 V for Li plating).

The temporal evolution of the phase interface, driven by the interfacial free energy and the electrode reaction affinity^51,52^, was given by a combination of non-conserved Allen-Cahn equation and Butler–Volmer equation

12 $$\frac{\partial \xi }{\partial t}=-{L}_{\sigma }\left[{g}^{{\prime} }\left(\xi \right)-\kappa {\nabla }^{2}\xi \right]-{L}_{\eta }{h}^{{\prime} }\left(\xi \right)\left\{\exp \left[\frac{{\alpha }_{a}F{\eta }_{a}}{{RT}}\right]-{\widetilde{c}}_{+}\exp \left[\frac{-{\alpha }_{c}F{\eta }_{a}}{{RT}}\right]\right\}.$$

Where ${L}_{\sigma }$is the interfacial mobility, ${L}_{\eta }$ is the reaction constant, ${\eta }_{a}$ is the activation overpotential and $g({{{{{\rm{\xi }}}}}})$ is an arbitrary double well function, expressed by $g\left(\xi \right)=W{\xi }^{2}{(1-\xi )}^{2}$. *W* in the equation is proportional to the barrier height, suggesting the amount of energy required for transformation from liquid phase to solid phase. $h(\xi )$ is an interpolation function, defined as $h\left(\xi \right)={\xi }^{3}(6{\xi }^{2}-15\xi+10)$.

The Li^+^ transport during the electro-deposition process was determined with a modified Nernst-Planck equation. The solid lithium was assumed to be immobile without diffusion, additionally, the effects of electron transport were neglected.

13 $$\frac{\partial {\widetilde{c}}_{+}}{\partial t}=\nabla \cdot \left[{D}^{{eff}}\left(\xi \right)\nabla {\widetilde{c}}_{+}+\frac{{D}^{{eff}}\left(\xi \right){\widetilde{c}}_{+}}{{RT}}F\nabla \varphi \right]-\frac{{c}_{s}}{{c}_{0}}\frac{\partial \xi }{\partial t}.$$

Where ${D}^{{eff}}$ is the effective Li^+^ diffusivity, determined by an interpolation function of the diffusion coefficient of Li^+^ in both the electrode and electrolyte^53^:

14 $${D}^{{eff}}={D}^{s}h\left(\xi \right)+{D}^{l}\left(1-h\left(\xi \right)\right).$$

Where ${D}^{s}$ is the Li^+^ diffusivity in the electrode.

Poisson equation was solved under the electroneutrality assumption to determine the spatial distribution of the electrical overpotential $\varphi$.

15 $$\nabla \cdot \left({\sigma }^{{eff}}\left(\xi \right)\nabla \varphi \right)={nF}{c}_{s}\frac{\partial \xi }{\partial t}.$$

Where the effective conductivity was modified by conductivity of the electrolyte ${\sigma }^{e}$ and the electrode ${\sigma }^{s}$:

16 $${\sigma }^{{eff}}={\sigma }^{s}h\left(\xi \right)+{\sigma }^{l}\left(1-h\left(\xi \right)\right).$$

Gas-evolving electrode surface structures were reconstructed by adding bubble domains into the phase-field model geometry. An implicit time integration was used for transient-time simulation, with a time step of △t = 0.2 s. Dirichlet boundary conditions were applied to solve the equations (13) and (15). The overpotential on the electrode-electrode interface and the boundary Li^+^ concentration were set at 0.10 V and 1.0 M, respectively. The dendrite’s areas under different electrode conditions were calculated by integrating $\xi$ in the whole simulation domain. Table S3 lists the physical property parameters and their normalized values of simulations.

## Supplementary information

Supplementary Information

## Data Availability

The datasets generated during and/or analyzed during the current study are available from the corresponding author on reasonable request.
