# Supplementary material for: Gas induced formation of inactive Li in rechargeable lithium metal batteries
Source: Nat Commun. 2023 Jan 12;14:177. doi: 10.1038/s41467-022-35779-0 (PMC9837134; doi:10.1038/s41467-022-35779-0)
Supplement: Supplementary file 1 — Supplementary Information [file 41467_2022_35779_MOESM1_ESM.pdf]

Supplementary information

Gas induced formation of inactive Li in rechargeable lithium metal batteries

Yuxuan Xiang[1,2,†], MingMing Tao[1,†], Xiaoxuan Chen[1,†], Peizhao Shan[1], Danhui Zhao[1], Jue Wu[1], Min Lin[1], Xiangsi Liu[1], Huajin He[1], Weimin Zhao[2], Yonggang Hu[1], Junning Chen[1], Yuexing Wang[3], Yong, Yang[1*]

[1]State Key Laboratory for Physical Chemistry of Solid Surfaces, Collaborative Innovation Center of Chemistry for Energy Materials and Department of Chemistry, College of Chemistry and Chemical Engineering, Xiamen University, Xiamen 361005, China.

[2] School of Engineering, Westlake University, Hangzhou, Zhejiang 310030, China.

[3]College of Chemical Engineering and Safety, Binzhou University, Binzhou 256603, PR China

[4]Institute of Electronic Engineering, China Academy of Engineering Physics, Mianyang 621999, China

This PDF file includes:

Supplementary Note 1

Figure S1-Figure S16

Table S1- Table S3

References (1- 21)

Supplementary Note 1

The Gibbs free energy calculation

$$C_2H_4(gas) + 6Li^0(crystal) \rightarrow 4LiH(crystal) + Li_2C_2(crystal)$$

$$\Delta G_f^\theta(Li) = 0\ KJ/mol$$

$$\Delta \mathrm{f} G_f^\theta(C_2H_4) = 68.15\ KJ/mol$$

$$\Delta G_f^\theta(LiH) = -68.447\ KJ/mol$$

$$\Delta G_f^\theta(Li_2C_2) = -56.11\ KJ/mol$$

$$\Delta G_r^\theta = 4 \times \Delta G_f^\theta(LiH) + \Delta G_f^\theta(Li_2C_2) - 6 \times \Delta G_f^\theta(Li) - \Delta G_f^\theta(C_2H_4) = \mathbf{-398.049\ \boldsymbol{KJ/mol}}$$

$$H_2(gas) + 2Li^0(crystal) \rightarrow 2LiH(crystal)$$

$$\Delta G_r^\theta = 2 \times \Delta G_f^\theta(LiH) - 2 \times \Delta G_f^\theta(Li) - \Delta G_f^\theta(H_2) = \mathbf{-136.894\ \boldsymbol{KJ/mol}}$$

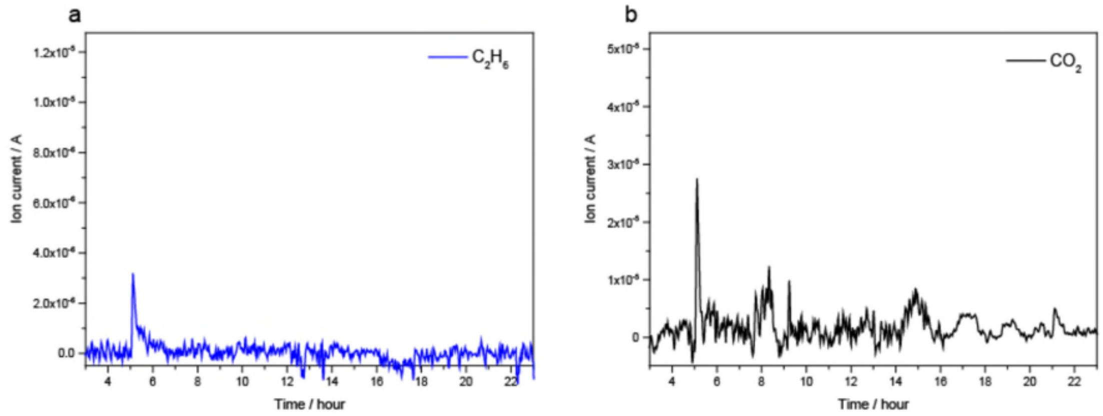

Figure S1| Evolution of gas species in Cu||$LiFePO_4$ batteries using 1M $LiPF_6$/EC:EMC electrolyte. (a) C_2H_6 evolution. **(b)** CO_2 evolution.

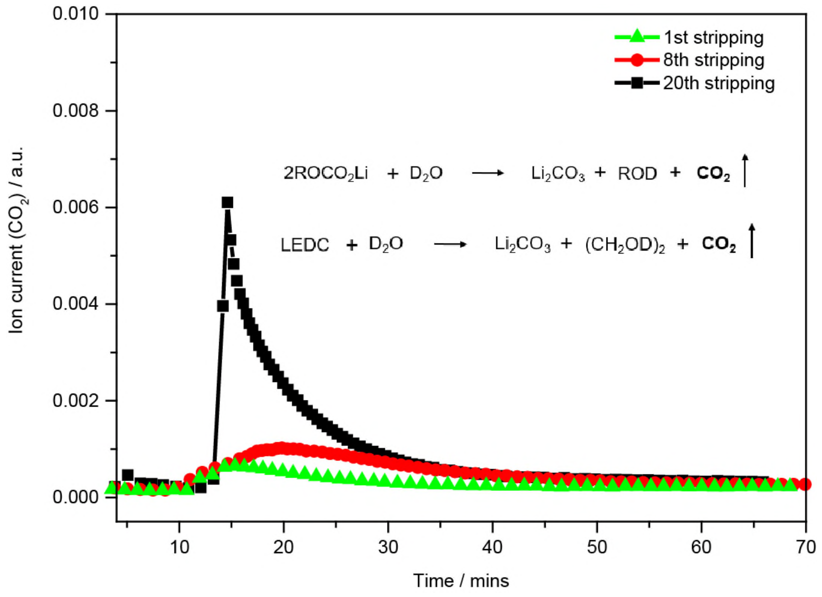

Figure S2| The evolution of CO_2 (the reactions products of LEDC/$ROCO_2Li$ with D_2O) varied with cycle number when using baseline electrolytes.

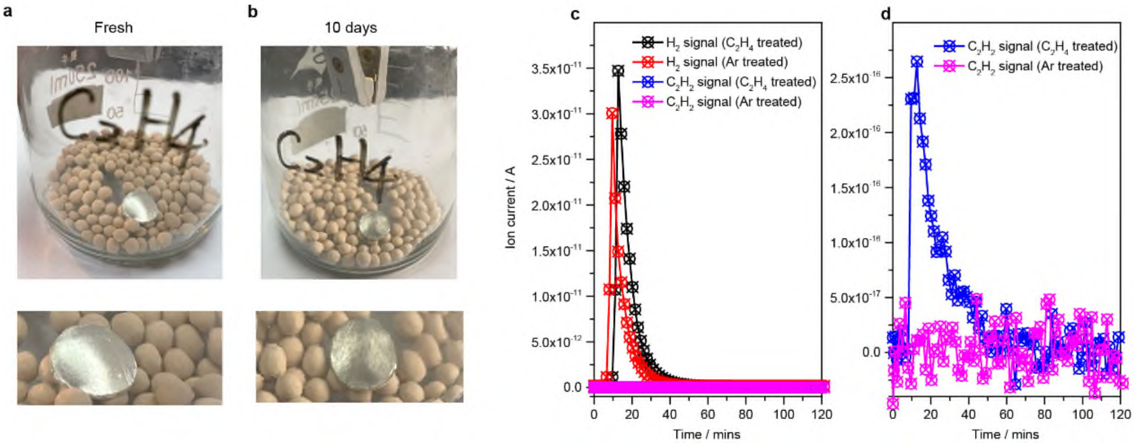

Figure S3| The reaction between Li metal with ethylene. (a) The fresh Li metal foil and **(b)** Li metal stored in C_2H_4 atmosphere for ten days. **(c)** The mass spectrometry titration results of surface species scratched from Li metal foils, which stored in C_2H_4 and Argon atmosphere for ten days respectively. m/z=2 (H_2) and m/z=26 (C_2H_2) signals are monitored. **(d)** The enlarged view of trace signals of C_2H_2.

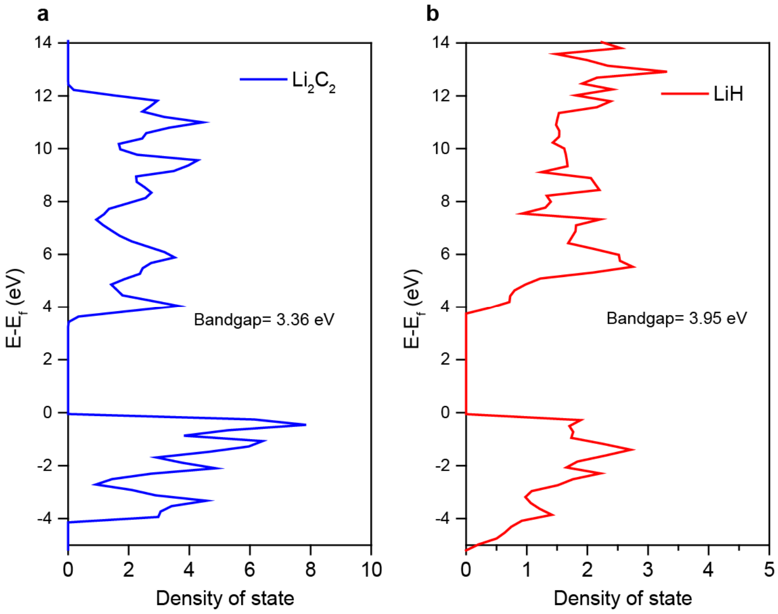

Figure S4| Density of state for (a) Li_2C_2 and **(b)** LiH.

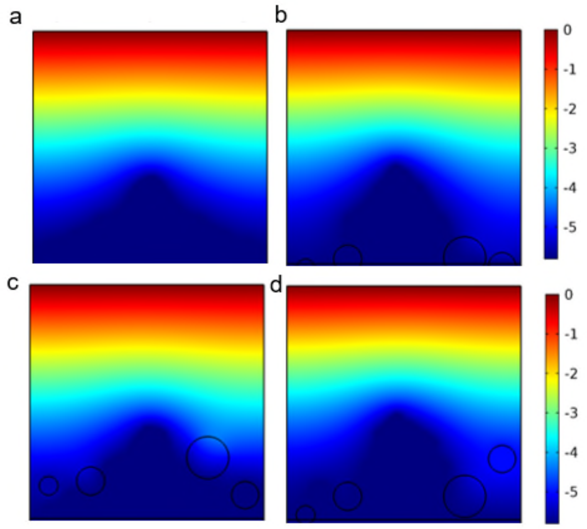

Figure S5| The distribution of electric field with bubbles (black cycle) formed at different locations.

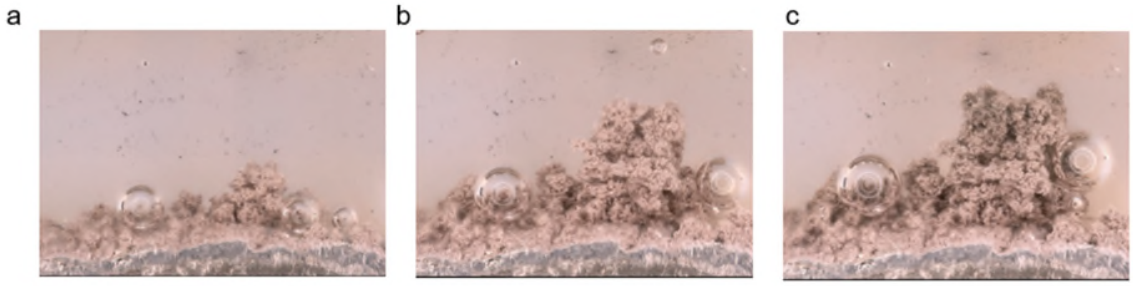

Figure S6| The morphological evolution of deposited sodium metal with the presence of bubbles as observed by optical microscopy. Copy right by ACS[1].

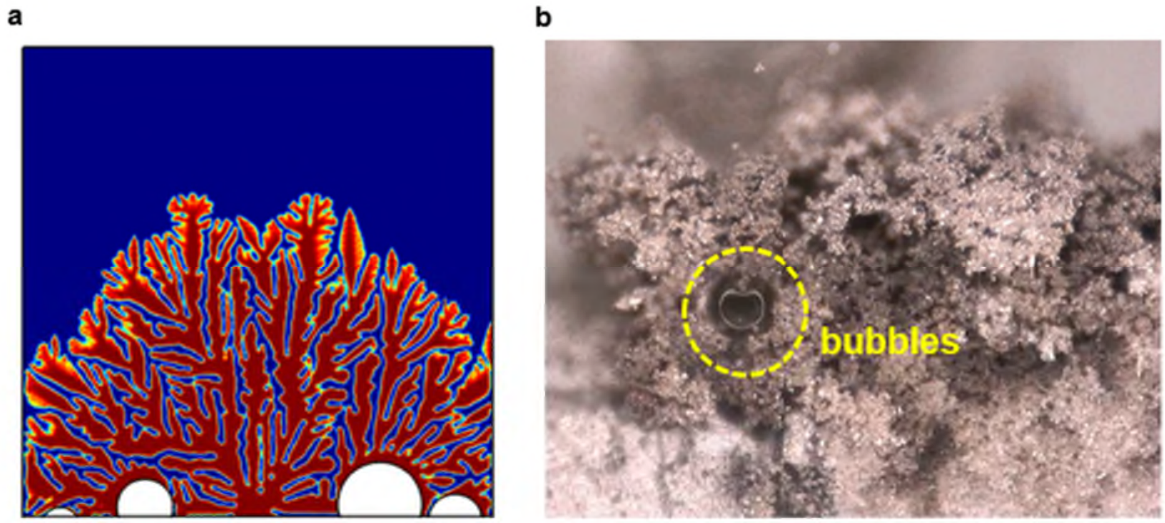

Figure S7| The formation of gas bubbles underneath the mossy type deposited metal is observed by (a) simulation results and **(b)** optical microscopy[1]. Copyright by ACS.

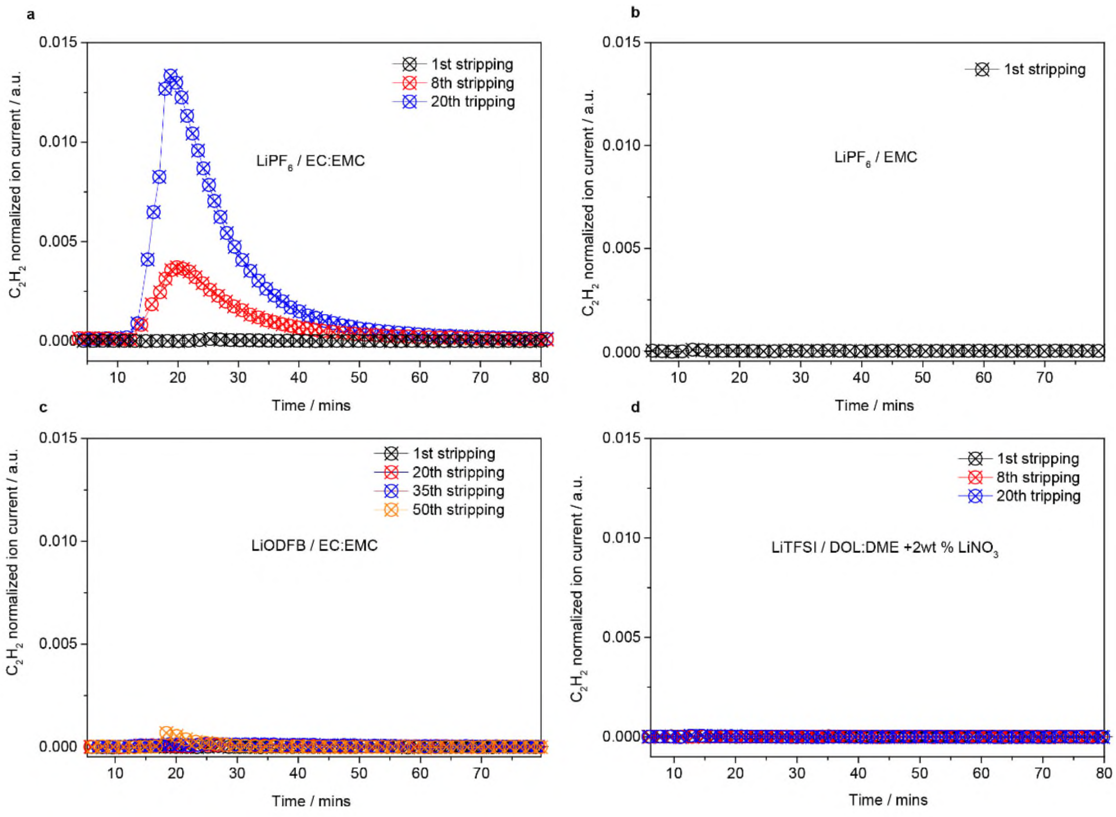

Figure S8| The evolution of C_2H_2 gas (the reactions products of Li_2C_2 with H_2O) varied with cycle number in different electrolyte systems. (a) 1M $LiPF_6$ / EC: EMC **(b)** 1M $LiPF_6$ /EMC **(c)** 1M LiODFB/EC: EMC and **(d)** 1M LiTFSI/ DOL: DME+2 wt% $LiNO_3$.

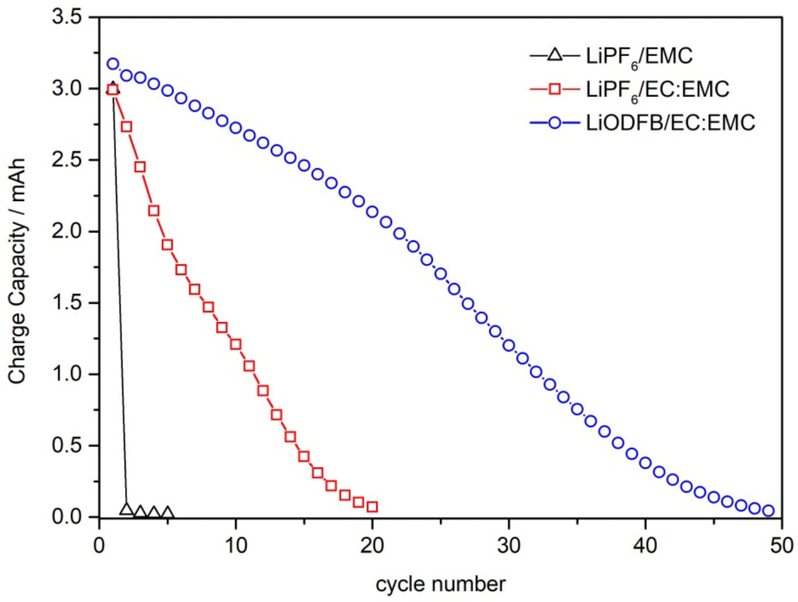

Figure S9| The charge capacity of Cu||$LiFePO_4$ cells varies with cycle time using 1M $LiPF_6$/EMC, 1M $LiPF_6$/EC: EMC (baseline) and 1M LiODFB/EC: EMC (LiODFB) electrolytes

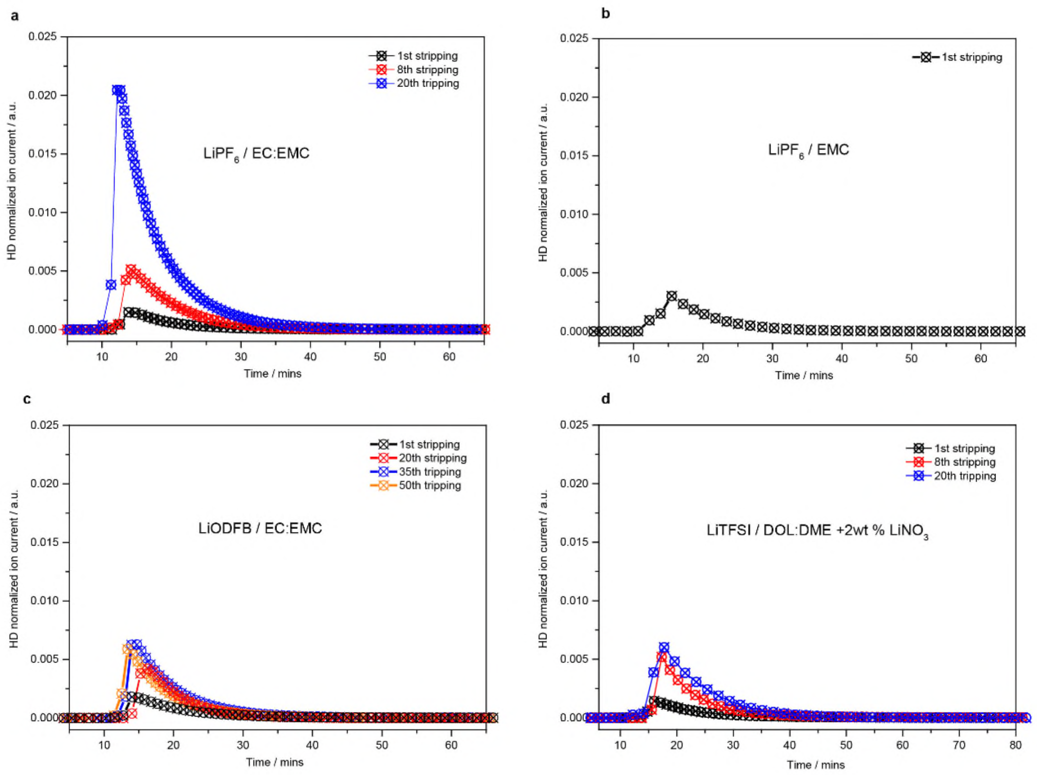

Figure S10| The evolution of HD (the reactions products of LiH with D_2O) varied with cycle number in different electrolyte systems. (a) 1M $LiPF_6$ / EC: EMC **(b)** 1M $LiPF_6$ /EMC **(c)** 1M LiODFB/EC: EMC and **(d)** 1M LiTFSI/ DOL:DME+2wt%$LiNO_3$.

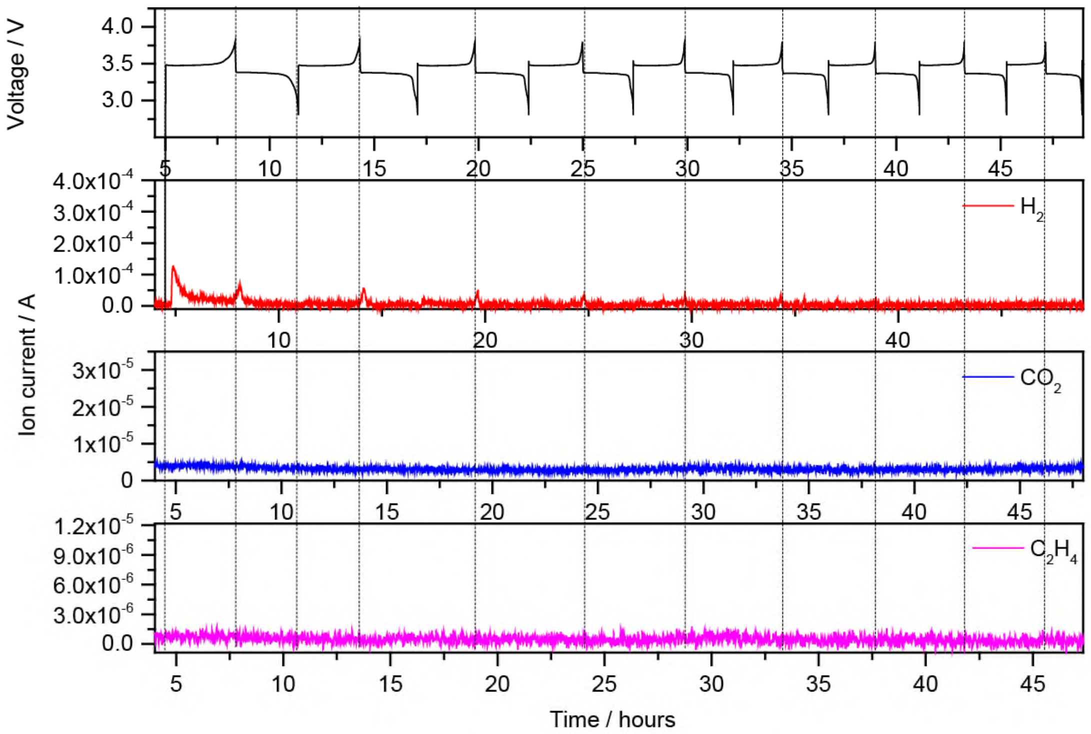

Figure S11| The evolution of gas species in Cu||LiFePO$_4$ batteries using 1M LiODFB/EC: EMC electrolyte. Voltage profile and the corresponding operando mass spectrometry results of H_2 (m/z=2), CO_2 (m/z=44) and C_2H_4 (m/z=26).

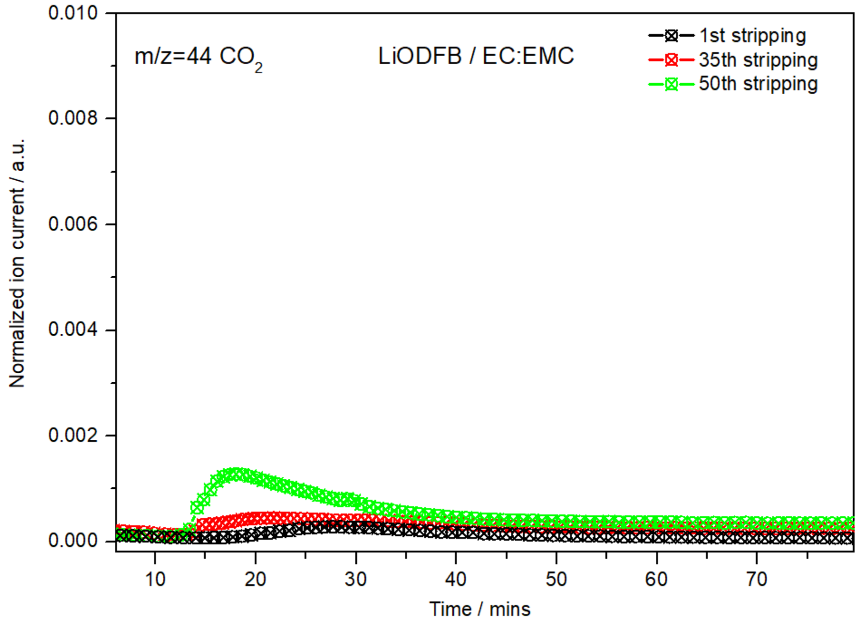

Figure S12| The evolution of CO_2 (the reactions products of LEDC with D_2O) varied with cycle number in LiODFB / EC: EMC electrolyte.

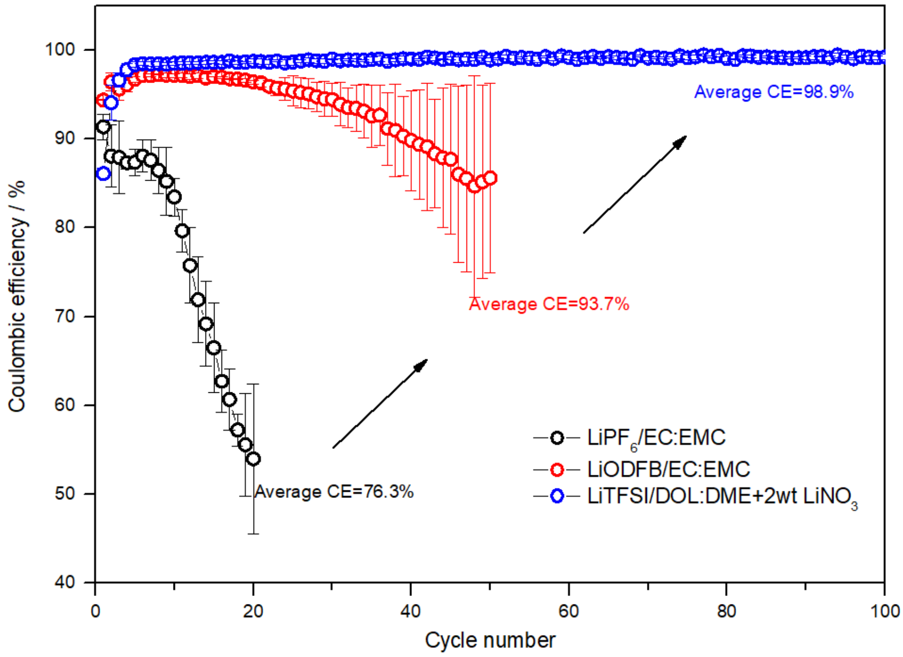

Figure S13| The coulombic efficiency of $LiFePO_4$||Cu batteries using different electrolytes. The error bars come from three independent experiments.

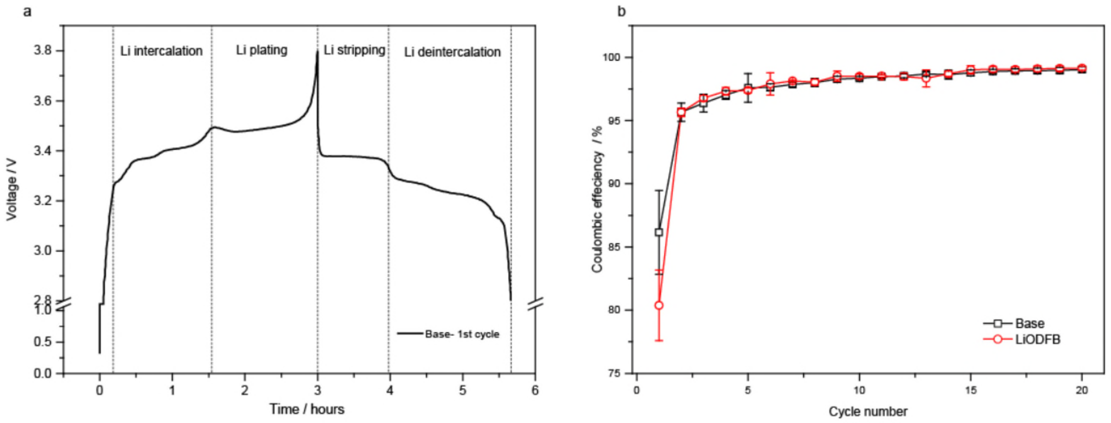

Figure S14| The electrochemical performance of LiODFB-based electrolyte and baseline electrolyte in $LiFePO_4$||Graphite full cells (N/P=0.73). (a) The first cycle performance. **(b)** Average Coulombic efficiency. The error bars come from three independent experiments.

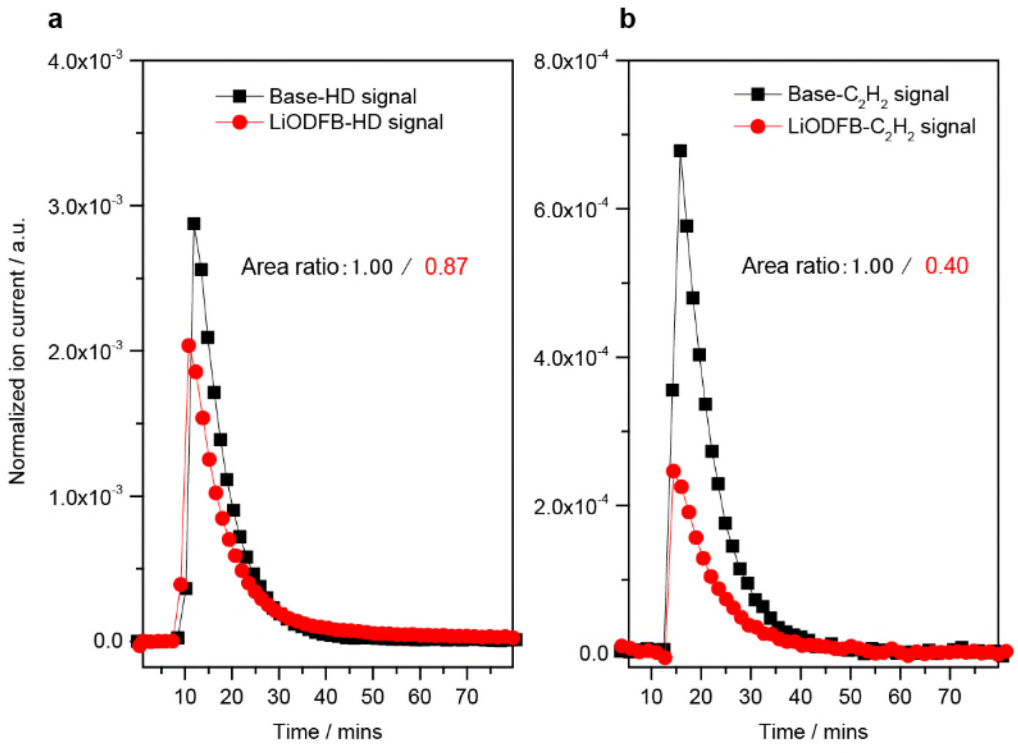

Figure S15| Mass spectrometry titration results of inactive Li formed on graphite after 20 cycles. (a) HD and **(b)** C_2H_2 signal for baseline electrolyte and LiODFB electrolyte.

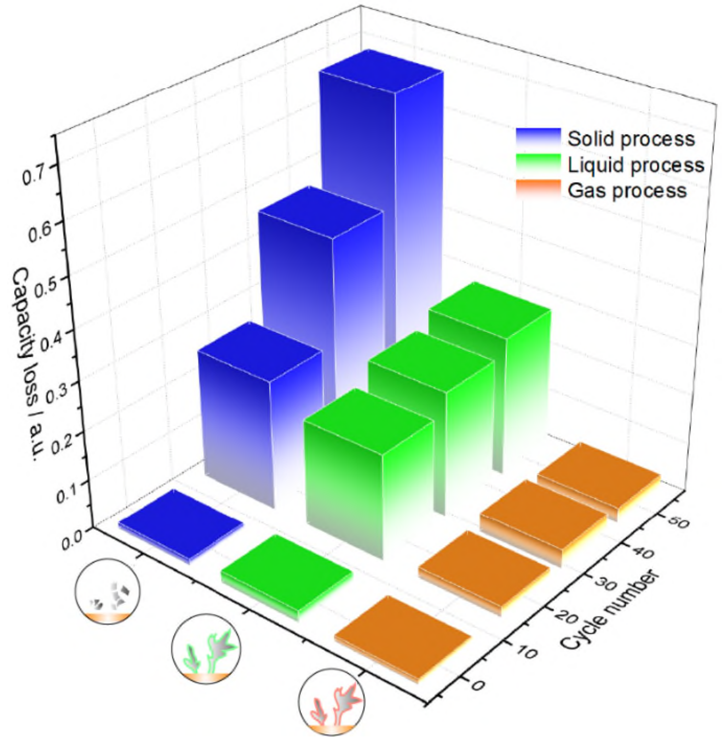

Figure S16| The distribution of inactive Lithium formed in the LiODFB / EC: EMC electrolyte.

Table S1.

A summary of the literature reporting the existence of Li_2C_2

Year published	Detected signal	Technique	Electrolyte	Anode	Ref
2013	C≡C bond	Raman	$LiPF_6$ in EC:DEC	Li metal	[2]
2016	C≡C bond	Raman	$LiPF_6$ in EC:EMC	Li metal	[3]
2016	Li_2C_2 (283 eV)	XPS	LiFSI,$LiPF_6$,LiTFSI,LiFTFSI in EC:DMC	Graphite	[4]
2019	C≡C bond	Surface enhanced Raman scattering	$LiPF_6$ in EC:EMC	Graphite	[5]
2020	C_2H_2 (m/z=26)	Mass spectrometry titration	$LiPF_6$ in EC:EMC	Graphite	[6]
2021	Li_2C_2 (282.2 eV)	XPS	$LiPF_6$ in EC:DMC	Li metal	[7]

Table S2.

The energy barrier of Li migration and the band gap of commonly reported organic SEI species.

SEI components	Energy barrier of Li migration / eV	Band gap / eV
LiF	0.729 eV[8]	8.72 eV
Li_2O	0.152 eV[8]	4.992 eV
Li_2CO_3	0.227-0.491 eV[8]	5.092 eV
LiH	/	3.95 eV
Li_2C_2	0.79eV[9]	3.36 eV

Table S3.

Phase-field simulation parameters and their normalized values

Parameter	Symbol	Real Value	Normalized Value	Reference
Interfacial mobility	L_σ	2.5×10^{-6} m^3 J^{-1} s^{-1}	1.5×10^4	[10,11]
Reaction constant	L_η	1.0 s^{-1}	2.3×10^3	[10-12]
Gradient energy coeff.	κ_0	5×10^{-5} J/m	7.7×10^{-3}	[10]
Barrier height	W	3.75×10^5 J m^{-3}	0.5	[10,13]
Diffusion coeff. In electrode	D^s	7.5×10^{-13} m^2 s^{-1}	30	[10,13,14]
Diffusion coeff. In electrolyte	D^l	7.5×10^{-10} m^2 s^{-1}	30	[10,13,14]
Conductivity in electrode	σ^s	1.0×10^7 S m^{-1}	1.0×10^9	[10,12]
Conductivity in electrolyte	σ^l	1.0 S m^{-1}	1.0×10^2	[10,12]
Molar gas constant	R	8.314 J (mol K)$^{-1}$	8.314	/
Reaction temperature	T	298 K	298	/
Faraday's constant	F	96485 C mol^{-1}	96485	/
Anodic charge-transfer coeff.	α_a	0.5	0.5	[15,16]
Cathodic charge-transfer coeff.	α_c	0.5	0.5	[15,16]
Mode of anisotropy	ω	4	4	[17-19]
Strength of anisotropy	Ω	0.05	0.05	[17-19]
Li^+ concentration in liquid	c_0	1.0×10^3 mol m^{-3}	1.0×10^3	[20,21]
Li atom concentration in metal	c_s	7.69×10^4 mol m^{-3}	7.69×10^4	[20,21]

References

1 Rodriguez, R. *et al.* In Situ Optical Imaging of Sodium Electrodeposition: Effects of Fluoroethylene Carbonate. *ACS Energy Lett.* **2**, 2051-2057 (2017).

2 Schmitz, R. *et al.* SEI investigations on copper electrodes after lithium plating with Raman spectroscopy and mass spectrometry. *J. Power Sources* **233**, 110-114 (2013).

3 Tang, S. *et al.* An electrochemical surface-enhanced Raman spectroscopic study on nanorod-structured lithium prepared by electrodeposition. *Journal of Raman Spectroscopy* **47**, 1017-1023 (2016).

4 Eshetu, G. G. *et al.* In-Depth Interfacial Chemistry and Reactivity Focused Investigation of Lithium–Imide- and Lithium–Imidazole-Based Electrolytes. *ACS Applied Materials & Interfaces* **8**, 16087-16100 (2016).

5 Fonseca Rodrigues, M.-T. *et al.* Lithium Acetylide: A Spectroscopic Marker for Lithium Deposition During Fast Charging of Li-Ion Cells. *ACS Applied Energy Materials* **2**, 873-881 (2019).

6 McShane, E. J. *et al.* Quantification of Inactive Lithium and Solid–Electrolyte Interphase Species on Graphite Electrodes after Fast Charging. *ACS Energy Lett.* **5**, 2045-2051 (2020).

7 Menkin, S. *et al.* Toward an Understanding of SEI Formation and Lithium Plating on Copper in Anode-Free Batteries. *J. Phys. Chem. C* (2021).

8 Chen, Y. C., Ouyang, C. Y., Song, L. J. & Sun, Z. L. Electrical and Lithium Ion Dynamics in Three Main Components of Solid Electrolyte Interphase from Density Functional Theory Study. *J. Phys. Chem. C* **115**, 7044-7049 (2011).

9 Ruprecht, B., Billetter, H., Ruschewitz, U. & Wilkening, M. Ultra-slow Li ion dynamics in Li2C2—on the similarities of results from7Li spin-alignment echo NMR and impedance spectroscopy. *J. Phys.: Condens. Matter* **22**, 245901 (2010).

10 Chen, L. *et al.* Modulation of dendritic patterns during electrodeposition: A nonlinear phase-field model. *Journal of Power Sources* **300**, 376-385 (2015).

11 Gao, L. & Guo, Z. Phase-field simulation of Li dendrites with multiple parameters influence. *Computational Materials Science* **183** (2020).

12 Yan, H. H., Bie, Y. H., Cui, X. Y., Xiong, G. P. & Chen, L. A computational investigation of thermal effect on lithium dendrite growth. *Energy Convers. Manage.* **161**, 193-204 (2018).

13 Yurkiv, V., Foroozan, T., Ramasubramanian, A., Shahbazian-Yassar, R. & Mashayek, F. Phase-field modeling of solid electrolyte interface (SEI) influence on Li dendritic behavior. *Electrochimica Acta* **265**, 609-619 (2018).

14 Gao, L. T., Huang, P., Feng, J., Zhu, R. & Guo, Z. S. In Situ Characterization and Phase‐Filed Modeling of the Interaction between Dendrites and Gas Bubbles during an Electrochemical Process. *ChemElectroChem* **8**, 2881-2887 (2021).

15 Shriram Santhanagopalana, C. L., Ralph E. White. Mathematical Modeling of Lithium Ion Batteries. *ECS Transactions* **16**, 81-90 (2008).

16 Sankarasubramanian, S. & Krishnamurthy, B. A capacity fade model for lithium-ion batteries including diffusion and kinetics. *Electrochimica Acta* **70**, 248-254 (2012).

17 Mu, W., Liu, X., Wen, Z. & Liu, L. Numerical simulation of the factors affecting the growth of lithium dendrites. *Journal of Energy Storage* **26** (2019).

18 Kobayashi, R. Modeling and numerical simulations of dendritic crystal growth. *Physica D: Nonlinear Phenomena* **63**, 410-423 (1993).

19 Kobayashi, R. A Numerical Approach to Three-Dimensional Dendritic Solidification. *Experimental Mathematics* **3**, 59-81 (1994).

20 Shen, X., Zhang, R., Shi, P., Chen, X. & Zhang, Q. How Does External Pressure Shape Li Dendrites in Li Metal Batteries? *Advanced Energy Materials* **11** (2021).

21 Rui Zhang, X. S., Xin-Bing Cheng, Qiang Zhang. The dendrite growth in 3D structured lithium metal anodes: Electron or ion transfer limitation? *Energy Storage Materials* **23**, 556–565 (2019).
